# Hiding behind ‘innovation’: the case for regulated risk assessment in surgery

**DOI:** 10.1093/bjs/znac347

**Published:** 2022-10-17

**Authors:** Jonathan Ives, Giles Birchley, Richard Huxtable, Jane Blazeby

**Affiliations:** Centre for Ethics in Medicine, NIHR Bristol Biomedical Research Centre, Bristol Medical School, University of Bristol, Bristol, UK; Centre for Ethics in Medicine, NIHR Bristol Biomedical Research Centre, Bristol Medical School, University of Bristol, Bristol, UK; Centre for Ethics in Medicine, NIHR Bristol Biomedical Research Centre, Bristol Medical School, University of Bristol, Bristol, UK; Centre for Surgical Research, NIHR Bristol Biomedical Research Centre, Bristol Medical School, University of Bristol, Bristol, UK

There have been numerous recent high-profile cases of ‘surgical innovation’ gone wrong, including vaginal mesh^1^, the da Vinci Robot^2^, and synthetic tracheas^3^. All were new developments in surgery that failed to reach their potential to benefit patients and surgeons, and instead caused harm. There is now urgent interest in how surgical innovation should be regulated.

Surgical innovation has the potential to bring significant benefit to patients, and innovator surgeons are historically heralded as inspirational leaders, and courageous risk-takers^4^, who develop more efficient and more effective procedures and devices that improve patient outcomes.

Currently, in the UK, surgeons are able to modify procedures, introduce procedures and devices, or use existing devices for new purposes with very little, if any, oversight. Guidance from the National Institute for Health and Care Excellence (NICE) is not followed^5^. Research itself is heavily regulated, and many UK hospitals have New Procedure Committees or Clinical Effectiveness Committees (or such like), providing an alternative ‘research approval’ process. They, in theory, monitor and authorize new procedures and new uses of devices^6,7^. Yet, if surgeons do not identify their practice as ‘research’, or do not self-identify as doing something ‘new’, these mechanisms fail, and practice goes unregulated and unmonitored.

Compounding this, there are limited reporting mechanisms for failed innovations, and existing ones are underused^5–7^. Consequently, innovations associated with harms/risks go unreported (unless picked up by regulators, the coroner, and/or the media) and future innovators cannot learn from them, meaning mistakes may be repeated. Mandated (cardiac surgery)^8^ and optional (e.g. arthroplasty) registries are designed to monitor standard (not innovative) procedures, and data capture is not routine. Although changes are occurring for the introduction of new devices, these are yet to be implemented. One challenge is defining what constitutes a new procedure, the associated risk, and what magnitude of modification/risk requires registration and/or regulation.

There is currently much focus on regulating surgical *innovation*^9,10^ to improve patient safety. We agree but, perhaps controversially, contend that the term ‘innovation’ should be eliminated.

## The case for eliminating ‘innovation’

Based on our research exploring the nature and meaning of surgical innovation, we argue the term ‘innovation’ is problematic^11^. ‘Innovation’ is a rich and value-laden concept, simultaneously conveying newness and benefit, and, as such, functions to characterize the ‘innovative’ as positive. ‘Innovation’ is supported and celebrated, and, if it is good to innovate, then, *ceteris paribus*, innovation is good. This rhetorical spin and ambiguity makes ‘innovation’ ill-suited for regulation or governance, not least because labelling a practice ‘innovation’ can hide a multitude of sins, both allowing the unconscientious surgeon to avoid regulatory scrutiny and misleading patients during informed consent processes, while staying *just* on the right side of truthfulness. Here, we outline our reasons for eliminating ‘innovation’ from our regulatory and informed consent lexicon, with renewed focus instead on newness and risk.

## Rationale for eliminating ‘innovation’

Any definition of ‘surgical innovation’ has to be specific and purposive, rather than generic^11^. Our own purpose is to improve the safe and transparent translation (including evaluation) of surgical innovation into practice. Key to determining safety and transparency is the stage of newness of the invasive procedure/use of device, which—we argue—in turn must be defined by what is known about its safety, efficacy, and effectiveness. If we are interested in regulation for safety and transparency, then the conceptual baggage that accompanies ‘innovation’ is irrelevant. Consequently, whether a procedure or device is ‘innovative’ has no bearing on how it should be regulated.

This association of ‘innovation’ with ‘newness’ is not itself new, and our work to this point follows colleagues at Macquarie^10^; but we diverge from that work in defining ‘newness’ not in terms of whether the surgeon recognizes a practice as new (which acts as a proxy for ‘innovative’), but in terms of how much is objectively known about the practice in terms of safety, efficacy, and effectiveness.

This moves away from conceptualizing newness as ‘ontological newness’ (whether it has been done before, ever, by anyone)—which leads to difficulties determining when a practice is new. Rather, ‘newness’ is conceptualized as ‘indexical newness’; that is, *newness in relation to the context in which it is occurring*.

This effectively defines ‘newness’ as a spectrum, the position on which a practice lands being based on what is known about the safety, efficacy, and effectiveness of a procedure/device in the context in which it is being used.

**Fig. 1:**
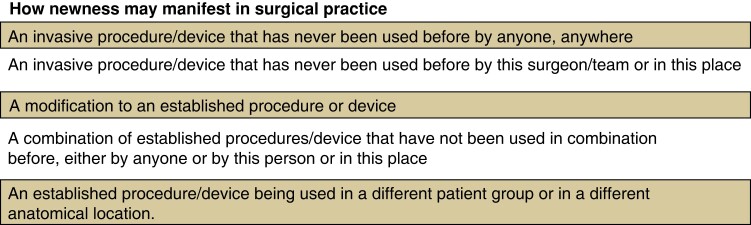
Manifestations of newness

Accordingly, ‘newness’ occurs whenever change is introduced to a surgeon’s practice. Any change introduces some level of uncertainty about safety, efficacy, and effectiveness, and the magnitude of newness is determined by the magnitude of uncertainty. ‘Newness’, therefore, manifests in various ways (*Fig. 1*).

All these manifestations introduce differences from the norm, and the degree of newness is determined by how much uncertainty about safety, efficacy, and effectiveness is introduced; sitting along a spectrum between ‘completely new’ and ‘not new at all’. For example, ‘completely new’ may be defined as ‘…insufficient reported knowledge to inform an assessment of safety, efficacy, and effectiveness.’

Conversely, ‘somewhat new’ may be defined as ‘…containing at least one different component/modification about which there is insufficient reported knowledge to inform assessment safety, efficacy, and effectiveness.’

**Fig. 2:**
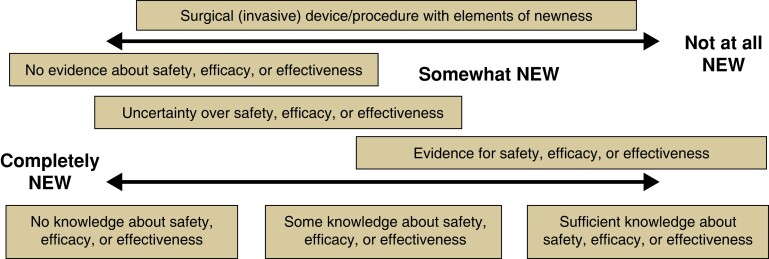
Spectrum of newness

‘Newness’ spans a continuum from ‘completely new’ to ‘not at all new’, with varying degrees of ‘somewhat new’ between the two (*Fig. 2*), each carrying different magnitudes of risk.

An invasive procedure may be introduced at any point along this spectrum and, regardless of its entry point, a risk assessment must be undertaken to ascertain how much risk is introduced. The ‘newer’ an intervention, the less evidence we will have about its risks and benefits. The less ‘new’ the intervention, the more evidence of its risks and benefits will be accessible.

A very new, high-risk procedure will require a different regulatory response to a somewhat new procedure introducing no/little risk, and each is likely to require a different kind of evaluation. Broadly, an intervention assessed to be completely new and high-risk may require registering, formal approval, and formal evaluation, similar to research. A partially new, low-risk intervention might require registering and reporting only. The most appropriate regulatory system will need developing, but what is clear is that whether or not the intervention in question is an ‘innovation’ is immaterial. Shifting the focus from ‘innovation’ towards what is known about safety, efficacy, and effectiveness will also help keep informed consent discussions clear, and focused on what is most important.

## The way forward

Improvements in surgery would be impossible without individuals who respond to surgical challenges creatively, pushing the boundaries and finding more cost-effective and efficient ways to help patients. However, the risks must be acknowledged and minimized.

The term ‘innovation’ carries an inherently positive rhetoric and ambiguity, making it unsuited for use in informed consent processes, and too unclear to be useful for regulation and governance.

We support experimentation and development, and encourage surgeons to embrace new and untrodden paths. To facilitate the safe and transparent translation of new invasive procedures/devices into practice we propose the elimination of ‘innovation’ from our informed consent and governance lexicon, in favour of the much clearer language of newness and risk.
